# Canadian multidisciplinary expert consensus on the use of biologics in upper airways: a Delphi study

**DOI:** 10.1186/s40463-023-00626-9

**Published:** 2023-04-24

**Authors:** Andrew V. Thamboo, Melissa Lee, Mohit Bhutani, Charles Chan, Yvonne Chan, Ken R. Chapman, Christopher J. Chin, Lori Connors, Del Dorscheid, Anne K. Ellis, Richard M. Gall, Krystelle Godbout, Arif Janjua, Amin Javer, Shaun Kilty, Harold Kim, Gordon Kirkpatrick, John M. Lee, Richard Leigh, Catherine Lemiere, Eric Monteiro, Helen Neighbour, Paul K. Keith, George Philteos, Jaclyn Quirt, Brian Rotenberg, Juan C. Ruiz, John R. Scott, Doron D. Sommer, Leigh Sowerby, Marc Tewfik, Susan Waserman, Ian Witterick, Erin D. Wright, Cory Yamashita, Martin Desrosiers

**Affiliations:** 1 Division of Otolaryngology-Head and Neck Surgery, Department of Surgery, St. Paul Sinus Center, University of British Columbia, 2600-1081 Burrard Street, Vancouver, BC V6Z 1Y6 Canada; 2 Department of Respirology, University of Alberta, Edmonton, AB Canada; 3 Department of Medicine, University of Toronto, Toronto, ON Canada; 4 Department of Otolaryngology-Head and Neck Surgery, University of Toronto, Toronto, ON Canada; 5 Division of Otolaryngology-Head and Neck Surgery, Department of Surgery, Dalhousie University, Halifax, NS Canada; 6 Department of Medicine, Dalhousie University, Halifax, NS Canada; 7 Department of Medicine, University of British Columbia, Vancouver, BC Canada; 8 Division of Allergy and Immunology, Department of Medicine, Queen’s University, Kingston, ON Canada; 9 Department of Otolaryngology-Head and Neck Surgery, University of Manitoba, Winnipeg, MB Canada; 10 Department of Medicine, Laval University, Quebec City, QC Canada; 11 Department of Otolaryngology-Head and Neck Surgery, The University of Ottawa and The Ottawa Hospital, Ottawa, ON Canada; 12 Division of Clinical Immunology and Allergy, Department of Medicine, Western University, London, ON Canada; 13 Division of Clinical Immunology and Allergy, Department of Medicine, McMaster University, Hamilton, ON Canada; 14 Division of Respiratory Medicine, University of British Columbia, Vancouver, BC Canada; 15 Department of Medicine, University of Calgary, Calgary, AB Canada; 16 Department of Medicine, CIUSS du Nord de l’île de Montreal, Université de Montreal, Montreal, QC Canada; 17 Lakeridge Health, Ajax, ON Canada; 18 Department of Otolaryngology-Head and Neck Surgery, Western University, London, ON Canada; 19 Division of Clinical Immunology and Allergy, University of Calgary, Calgary, AB Canada; 20 Division of Otolaryngology-Head and Neck Surgery, Department of Surgery, McMaster University, Hamilton, ON Canada; 21 Department of Otolaryngology-Head and Neck Surgery, McGill University, Montreal, QC Canada; 22 Division of Otolaryngology-Head and Neck Surgery, Department of Surgery, University of Alberta, Edmonton, AB Canada; 23 Department of Medicine, Western University, London, ON Canada; 24 Division of Otolaryngology-Head and Neck Surgery, Centre Hospitalier de l’Université de Montreal, Montreal, QC Canada

**Keywords:** Chronic rhinosinusitis, Chronic rhinosinusitis with nasal polyposis, Upper airway disease, Lower airway disease, Asthma, Biologics, Type 2 inflammation

## Abstract

**Background:**

Chronic rhinosinusitis with nasal polyposis (CRSwNP) often coexists with lower airway disease. With the overlap between upper and lower airway disease, optimal management of the upper airways is undertaken in conjunction with that of the lower airways. Biologic therapy with targeted activity within the Type 2 inflammatory pathway can improve the clinical signs and symptoms of both upper and lower airway diseases. Knowledge gaps nevertheless exist in how best to approach patient care as a whole. There have been sixteen randomized, double-blind, placebo-controlled trails performed for CRSwNP targeted components of the Type 2 inflammatory pathway, notably interleukin (IL)-4, IL-5 and IL-13, IL- 5R, IL-33, and immunoglobulin (Ig)E. This white paper considers the perspectives of experts in various disciplines such as rhinology, allergy, and respirology across Canada, all of whom have unique and valuable insights to contribute on how to best approach patients with upper airway disease from a multidisciplinary perspective.

**Methods:**

A Delphi Method process was utilized involving three rounds of questionnaires in which the first two were completed individually online and the third was discussed on a virtual platform with all the panelists. A national multidisciplinary expert panel of 34 certified specialists was created, composed of 16 rhinologists, 7 allergists, and 11 respirologists who evaluated the 20 original statements on a scale of 1–9 and provided comments. All ratings were quantitively reviewed by mean, median, mode, range, standard deviation and inter-rater reliability. Consensus was defined by relative interrater reliability measures—kappa coefficient ($\kappa$) value > 0.61.

**Results:**

After three rounds, a total of 22 statements achieved consensus. This white paper only contains the final agreed upon statements and clear rationale and support for the statements regarding the use of biologics in patients with upper airway disease.

**Conclusion:**

This white paper provides guidance to Canadian physicians on the use of biologic therapy for the management of upper airway disease from a multidisciplinary perspective, but the medical and surgical regimen should ultimately be individualized to the patient. As more biologics become available and additional trials are published we will provide updated versions of this white paper every few years.

**Graphical abstract:**

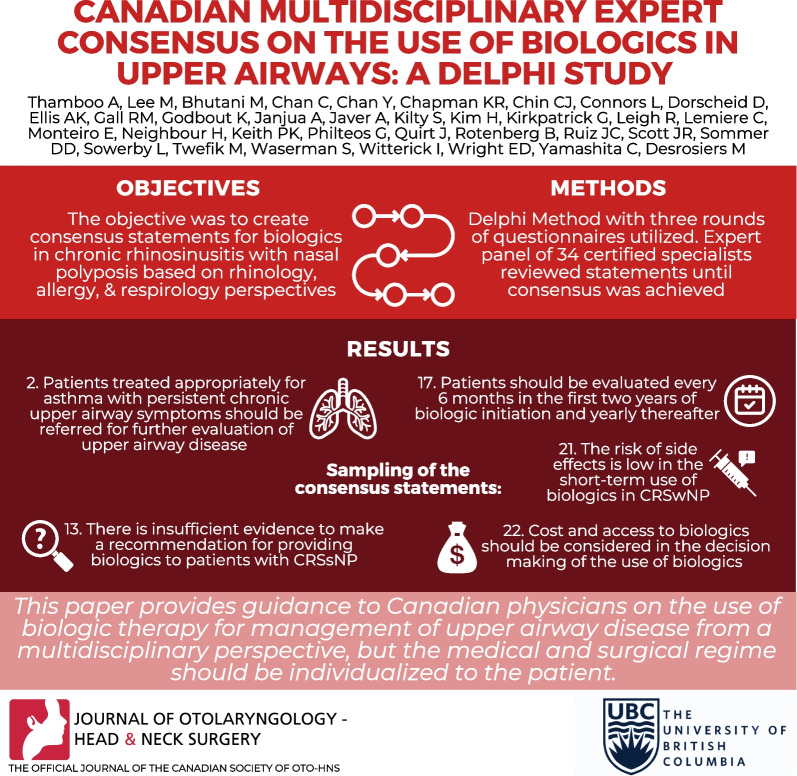

## Background

Chronic rhinosinusitis with nasal polyps (CRSwNP) is a disabling upper airway inflammatory disease that is characterized by significant patient morbidity resulting in exposure to long-term topical and systemic corticosteroids as well as surgical interventions. Moreover, many patients with CRSwNP suffer from comorbid lower airway disease such as asthma [1]. An improved understanding of the underlying disease pathophysiology of Type 2 inflammation, which is characterized by the presence of eosinophilic airway inflammation associated with IL-4, IL-5, IL-5Rα, IL-13 and circulating or local IgE, has led to new developments in medical management of CRSwNP that are aimed at modulating the Type 2 inflammatory response [2]. The results of clinical trials involving Type 2 inflammation indicate that biologic treatments with targeted activity within the Type 2 inflammatory pathway can improve the clinically relevant signs and symptoms of CRSwNP disease in patients who are medically and/or surgically recalcitrant (Table 1) [3–16]. This has resulted in the emergence of biologic monoclonal antibody agents as an adjunctive therapeutic modality for CRSwNP.

**Table 1:** Results of biologic randomized controlled trials for CRSwNP

Study	Treatment	Study sample size	Duration	Condition	Comorbidities	Study outcomes	Results
﻿Effect of Subcutaneous Dupilumab on Nasal Polyp Burden in Patients With Chronic Sinusitis and Nasal Polyposis: A Randomized Clinical Trial Bachert et al. [3]	﻿Dupilumab (anti-IL-4/IL-13)	N = 60 (Placebo n = 30, Treatment n = 30)	16 weeks (study endpoint)	CRSwNP refractory to intranasal corticosteroid therapy	Asthma: n = 35 Allergies: n = 38 Aspirin intolerance: n = 12 Placebo Asthma: n = 19 (63.3%) Aspirin sensitization: n = 9 (30%) Treatment Asthma: n = 16 (53.3%) Aspirin sensitization: n = 6 (20%)	﻿Endoscopic NPS, SNOT-22, VAS, NPIF, UPSIT, CT (LMK), FEV1, ACQ-5	Statistically significant improvement in SNOT-22 and UPSIT in the dupilumab group versus placebo Statistically significant difference of least squares mean change in bilateral endoscopic nasal polyp score and LMK CT total scores between the treatment and placebo group Statistically significant reduction of IgE, and plasma eotaxin-3 with dupilumab versus placebo *Asthma*: Dupilumab and mometasone improved lung function and asthma control by FEV1, ACQ-5, UPSIT, SNOT22
﻿Efficacy and safety of dupilumab in patients with chronic rhinosinusitis with nasal polyps: results from the randomized phase 3 SINUS-24 study Bachert et al. [4]	﻿Dupilumab (anti-IL-4/IL-13)	﻿N = 276 (Placebo n = 133, Treatment n = 143)	24 weeks (study endpoint) with 24 weeks follow-up	﻿CRSwNP	58% comorbid asthma 30% comorbid AERD	﻿VAS, SNOT-22, adverse events, patient-reported outcomes, ACQ- 6, total nasal polyp score, UPSIT, FEV1, LMK score, blood and serum markers	Dupilumab significantly improved nasal polyp score, LMK score, Snot- 22 score, patient reported nasal congestion, and UPSIT scores from baseline compared to placebo Asthma patients on dupilumab had improved lung function (FEV1) and ACQ-6 scores By week 24, significant decrease in NPS by 1.89 points and decrease congestion by 1.34 points was observed Week 4–8 showed significant improvement in NPS and congestion After stopping injections at 24 weeks, all metrics trended back towards baseline
﻿A randomized phase 3 study, SINUS-52, evaluating the efficacy and safety of dupilumab in patients with severe chronic rhinosinusitis with nasal polyps Han et al. (2019)^5^	﻿Dupilumab (anti-IL-4/IL- 13)	﻿N = 448 (Placebo n = 153, Treatment Q2W/Q4W n = 145, Treatment Q2W n = 150)	52 weeks (study endpoint)	﻿CRSwNP	60% comorbid asthma 27% comorbid AERD	﻿VAS, SNOT-22, adverse events, patient-reported outcomes, ACQ- 6, total nasal polyp score, UPSIT, FEV1, LMK score, blood and serum markers,	Dupilumab significantly improved nasal polyp score, LMK score, Snot- 22 score, patient reported nasal congestion, and UPSIT scores from baseline compared to placebo Asthma patients on dupilumab had improved lung function (FEV1) and ACQ-6 scores By week 24, significant dec NPS by 1.71 points and decrease in congestion by 1.25 points was observed When injections continued after 24 weeks, patients continued to see benefit and was greatest in q2w versus q4w By week 4–8, patients showed significant improvement in NPS and congestion In 2/3 patients, surgical procedure was no longer necessary
Dupilumab reduces opacification across all sinuses and related symptoms in patients with CRSwNP Bachert et al. [6]	Dupilumab (anti-IL-4/IL-13)	N = 60 (Placebo n = 30, Treatment n = 30)	16 weeks (study endpoint)	CRSwNP	Comorbid asthma Placebo: n = 19(63.3%) Treatment: N = 16(53.3%) Placebo n = 19 Treatment n = 16	zLMK, LMK score, bilateral endoscopic nasal polyp score, UPSIT, SNOT-22, VAS, patient reported symptoms of nasal congestion and/or obstruction	After 16 weeks, Dupilumab significantly decreased opacification across all sinuses measured using the LMK and zLMK scoring systems, and significantly improved nasal polyp score, SNOT-22 score, VAS score, and UPSIT score At baseline opacification measured by total LMK score correlated with other assessed outcomes but not at 16 weeks
A randomized, double-blind, placebo-controlled trial of anti-IgE for chronic rhinosinusitis Pinto et al. [7]	Omalizumab (anti-IgE)	N = 14 (Placebo n = 7, Treatment n = 7)	6 months (study endpoint)	CRSwNP	Asthma: All patients	﻿Snot-22, SF-36, nasal polyp size, CT scan opacification percentage, adverse events, NPIF, eosinophil count, UPSIT	No significant differences in polyp size, CT scan opacification percentage, SNOT-22 score, NPIF in the omalizumab group compared to placebo Improvement in UPSIT smell test score but not statistically significant and no significant differences in SF-36 except for the one domain, Vitality, between omalizumab and placebo group
﻿Omalizumab is effective in allergic and nonallergic patients with nasal polyps and asthma Gevaert et al. [8]	Omalizumab (anti-IgE)	﻿N = 24 (Placebo n = 8, Treatment n = 15)	﻿16 weeks (study endpoint)	﻿CRSwNP with asthma	﻿Asthma: All patients Allergies: n = 13 (placebo n = 7 (47%), treatment n = 6 (75%)) Aspirin intolerance: n = 12 Placebo 9 (53%), treatment = 4(50%)	﻿Disease symptom scores, adverse events, RSOM-31, AQLQ, SF-36, polyp size and total overall polyp score, LMK Score, FEV1 and PEF, and blood and serum markers	Significant reduction in polyp size, improvement in LMK scores in Omalizumab group after 16 weeks Significant decrease in symptom scores for Omalizumab group: nasal congestion, anterior rhinorrhea, loss of sense of smell, dyspnea Significant improvement in SF-36 of physical health, RSOM-31 of sleep and general symptoms and AQLQ after Omalizumab treatment No significant changes in blood and serum markers were observed at study endpoint No significant differences were observed in outcomes between allergic, non-allergic and AERD patients
Efficacy and safety of omalizumab in nasal polyposis: 2 randomized phase 3 trials (POLYP 1) Gevaert et al. [9]	Omalizumab (anti-IgE)	N = 138 (Placebo n = 66, Treatment n = 72)	24 weeks (study endpoint)	CRSwNP	Asthma Placebo: N = 32 (48.5%), treatment: N = 42 (58.3%) AERD: Placebo: N = 11 (16.7%), treatment N = 16 (22.2%)	endoscopic NPS, NCS, NOT-22, UPSIT, TNSS, % requiring rescue therapy, comorbid asthma, number of asthma exacerbations, AQLQ	Significant improvement in mean NPS and daily NCS at week 24 and as early as week 4, week 8 for UPSIT Comorbid asthma and AERD showed similar improvements in comparison to those without AERD Significant improvement in SNOT-22, UPSIT, TNSS and individual nasal symptoms were observed There was a 62.5% relative reduction in rescue steroid use in treatment group There was a reduced need for surgery and reduced number of asthma exacerbations observed after treatment
Efficacy and safety of omalizumab in nasal polyposis: 2 randomized phase 3 trials (POLYP 2) Gevaert et al. [9]	Omalizumab (anti-IgE)	N = 127 (Placebo n = 65, Treatment n = 62)	24 weeks (study endpoint) with 24 weeks follow-up	CRSwNP	Asthma: Placebo: n = 39 (60%), treatment: n = 38 (61.3%) AERD: Placebo: n = 21 (32.3%), treatment: n = 24 (38.7%)	endoscopic NPS, NCS, NOT-22, UPSIT, TNSS, % requiring rescue therapy, comorbid asthma, AQLQ	There was a significant improvement in mean NPS and daily NCS at week 24 and as early as week 4, week 8 for UPSIT Comorbid asthma and AERD showed similar improvements in comparison to those without AERD Significant improvement in SNOT-22, UPSIT, TNSS and individual nasal symptoms 62.5% patients demonstrated relative reduction in rescue steroid use, reduced need for surgery and reduced number of asthma exacerbations
Continued safety/efficacy of omalizumab in chronic rhinosinusitis with nasal polyps: an open-label extension study Gevaert et al. [16]	Omalizumab (anti-IgE)	N = 249 (Placebo n = 126, Treatment n = 123)	76 weeks (study endpoint, 24 weeks of follow-up after 52 weeks)	CRSwNP	Same patients from Polyp I and II	endoscopic NPS, NCS, NOT-22, UPSIT, TNSS, % requiring rescue therapy, comorbid asthma, AQLQ	Patients who received omalizumab in Polyp 1 or Poly 2 trials experienced continued improvement in NPS, NCS, and SNOT-22 through week 52 in comparison to placebo After omalizumab withdrawal at 52 weeks, all outcomes worsened/trended back to baseline, but remained below pre-treatment levels at week 76 Safety and efficacy profile of omalizumab same as previous studies
﻿Reduced need for surgery in severe nasal polyposis with mepolizumab: randomized trial Bachert et al. [10]	Mepolizumab (anti-IL-5)	﻿N = 105 (Placebo n = 51, Treatment n = 54)	25 weeks (study endpoint)	﻿CRSwNP	﻿ Asthma n = 82	﻿VAS, SNOT-22, adverse events, ﻿avoidance of surgery, endoscopic nasal polyp score, EQ-5D, Sniffin’ Sticks Screening-12, and lung function assessments	Significant improvement ﻿endoscopic nasal polyp score, all individual VAS symptom scores, and SNOT-22 score in the mepolizumab compared with placebo group The was no statistically significant difference in olfaction via Sniffin’ Sticks Screening-12, and lung function tests A reduction in blood eosinophil counts in the mepolizumab but not in the placebo After 25 weeks of dosing every 4 weeks, 5% patients required surgery compared to 30% of patients in the placebo group
﻿Mepolizumab, a humanized anti-IL-5 mAb, as a treatment option for severe nasal polyposis Gevaert et al. [11]	Mepolizumab (anti-IL-5)	N = 30 (Placebo n = 10, Treatment n = 20)	48 weeks (study endpoint)	﻿CRSwNP refractory to corticosteroid therapy	﻿Asthma: n = 23 Allergies: n = 14 Aspirin intolerance: n = 5	﻿Disease symptom scores, Adverse events, nasal polyp score, CT scan score, NPIF, blood and serum markers	Significant improvement in total polyp score and CT scan scores from baseline in the mepolizumab group compared to placebo No significant difference in disease symptoms scores or NPIF Significant reduction of blood eosinophil counts and serum ECP and serum IL-5Rα levels at week 8 in mepolizumab group Nasal IL-5Rα, IL-6, IL-1β, and MPO levels were significantly reduced in the mepolizumab group
Mepolizumab for CRSwNP (SYNAPSE): a randomised, double-blind, placebo-controlled phase 3 trial Han et al. [12]	Mepolizumab (anti-IL-5)	N = 407 (Placebo n = 201, Treatment n = 206)	52 weeks (study endpoint)	CRSwNP refractory to medical and surgical management	Asthma: Placebo: N = 149 (74%), treatment N = 140 (68%) AERD: Placebo: N = 63 (31%), treatment: N = 45 (22%)	NPS, nasal obstruction. SNPT22, Peak nasal inspiratory flow, UPSIT, blood samples, ECG	Mepolizumab treatment improved nasal polyp size, nasal obstruction VAS compared with placebo 41% of patients treated with mepolizumab required a course of antibiotics in comparison to 50% of patients in the placebo group Subgroup analyses of the coprimary endpoints suggested that the efficacy of mepolizumab is higher with higher baseline blood eosinophil count The risk of nasal surgery was lower in the mepolizumab group (9%) versus the placebo group (23%) Patients demonstrated relative reduction in rescue steroid use, reduced need for surgery and reduced number of asthma exacerbations
Efficacy of benralizumab in CRSwNP with nasal polyps: A randomized placebo-controlled trial (OSTRO) Bachert et al. [13]	Benralizumab (anti-IL-5/Ra)	N = 413 (Placebo n = 206, Treatment n = 207)	40 weeks (study endpoint) extended to 60 and 80 weeks for follow-up	CRSwNP refractory to ICS	Asthma: Placebo: n = 142 (68.6%), treatment: n = 59(29.1%) AERD Placebo: n = 62 (30%), treatment: n = 59 (29.1%)	NPS, NBS, SNOT22, DSS, LMS, UPSIT, SCS use, ACQ-6, Adverse events, ADA	There was a significant improvement in NPS and Nasal blockage score at week 40 in treatment group There were no significant improvements in SNOT22, time to first nasal polyp surgery and/or SCS use At week 40, a decrease in baseline ACQ-6 was observed for the treatment group and not the placebo group Treatment favoured benralizumab up to week 56 in comparison to placebo
A Phase II, multicenter, randomized placebo-controlled study of benralizumab, in patients with eosinophilic CRS Takabayashi [14]	Benralizumab (anti-IL-5/Ra)	N = 56 (Placebo n = 11, Single dose n = 22, q4w n = 23)	12 weeks (study endpoint) with 12 weeks follow-up	ECRS (eosinophilic CRSwNP)	Asthma: Placebo: n = 10 (90.9%), treatment one: n = 18(81.8%), treatment 2: n = 19(82.6%) AERD Placebo: n = 5(45.5%), treatment one: n = 6 (27.3%), treatment 2: n = 6 (26.1%)	NPS, LMK, Symptoms severity, blood eosinophil	There was no significant difference in change in nasal polyp score from baseline at week 12 There was a decrease in NPS of > 2 points in 42.2% eosinophilic CRS patients High blood eosinophil level was associated with improved response to biologic treatment (eosinophil count significantly decreased and remained at 0/uL up to week 8 with a single dose and week 12 with 3 doses)
Benralizumab effect on severe CRSwNP: A randomized double-blind placebo-controlled trial Tversky et al. [15]	Benralizumab (anti-IL-5/Ra)	N = 24 (Placebo n = 12, Treatment n = 12)	20 weeks (study endpoint) with 4 weeks follow-up	CRSwNP, refractory to medical and surgical management	Asthma: Placebo: n = 10 (83%), treatment: n = 12 (100%) AERD Placebo: n = 3 (25%), treatment: n = 8(67%)	Polyp score, CT, SNOT22, Sniffin’ Stick smell test	There were significant improvement in NP score observed in patients in the treatment group There was a dec in NP size in the treatment group, but was not found to be statistically significant The ratio of blood eosinophil count to allergen skin test positivity correlated with polyp reduction in the treatment group There was a 42% improvement in all outcomes observed in the treatment group

*CRSwNP* Chronic Rhinosinusitis with Nasal Polyposis, *CRSsNP* Chronic Rhinosinusitis without Nasal Polyposis, *NPIF* Nasal peak inspiratory flow, *Serum ECP* Serum Eosinophil cationic protein, *IL-5Rα *Interleukin-5 receptor α, *IL* Interleukin, *MPO* myeloperoxidase, *SNOT-22* Sino-Nasal Outcome Test-22, *SF-36* 36-Item Short Form Survey, *UPSIT* The University of Pennsylvania Smell Identification Test, *RSOM-31* 31-item Rhinosinusitis Outcome Measure, *AQLQ* Asthma Quality of Life Questionnaire, *FEV1* Forced Expiratory Volume, *PEF* Peak Expiratory Flow, *VAS* Visual Analogue Scale, *EQ-5D* Generic health- related quality of life questionnaire, *ACQ-6* 6-question Asthma Control Questionnaire, *ACQ-5* 5-question Asthma Control Questionnaire, *LMK* Lund- Mackay Score, *zLMK* Zinreich-modified Lund–Mackay Score

There is growing evidence to support the concept of the unified airway, which proposes that the respiratory system (upper and lower airways) functions as a single unit [1]. As such, pathological processes that occur in either the upper or lower airways share common pathophysiological mechanisms driving the disease endotype, of which Type 2 inflammation is the most prominent. Asthma often coexists in patients with CRSwNP and the presence of nasal polyps is associated with more severe asthma disease phenotype [17]. The management of CRS with comorbid asthma has been shown to be more difficult, leading to the increased use of oral corticosteroids for both polyp and/or asthma control, and increased need for revision surgery [18, 19]. Thus, the burden of disease is increased in patients with CRSwNP and comorbid asthma.

With the overlap between upper and lower airway disease, optimal management of the upper airways is undertaken in conjunction with that of the lower airways. Knowledge gaps nevertheless exist in how best to approach patient care as a whole. To that end, this white paper considers the perspectives of experts in various disciplines such as rhinology, allergy, and respirology across Canada, all of whom have unique and valuable insights to contribute on how to best approach patients with upper airway disease from a multidisciplinary perspective.

## Rationale for use of biologics in type 2 inflammatory disease

The cornerstone of the management of both CRSwNP consists of anti-inflammatory treatment with topical corticosteroids, with the goal of achieving both inflammation and optimal disease control [20]. When topical treatment is insufficient, short courses of oral corticosteroids are often used for symptom control [21]. Patients with refractory CRSwNP often undergo endoscopic sinus surgery[20]. Despite these management options, patients with CRSwNP can fail both medical and surgical interventions. In the past decade, more attention has been directed to the unified airway hypothesis and focusing on “treatable traits” [1]. Under this hypothesis, therapy is driven by patients’ individual disease-associated characteristics. Treatable traits in patients with CRSwNP with coexisting lower airway disease include asthma, smoking, allergy, occupational exposures, and mucociliary clearance deficits. Using treatable traits, therapies can be directed to an individual’s disease-associated characteristics [22].

In Canada, biologic agents have entered the market as therapeutic options for disease processes driven by Type 2 inflammatory pathways including severe allergic asthma, severe eosinophilic asthma, and atopic dermatitis [2]. Agents that are currently approved or under review for the treatment of CRSwNP target the Type 2 inflammatory pathway, notably interleukin IL-4, IL-5, IL-13, IL-5Rα, and IgE, and have been previously approved for use in asthma and/or atopic dermatitis [2]. All the studies that have been conducted to date have included patients with CRSwNP, asthma or atopic dermatitis. As of October 10, 2022, there have been 16 randomized, double-blind, placebo-controlled trials performed using biologics that target the aforementioned inflammatory mediators and one trial is currently underway that targets IgE in CRSwNP patients. The details of the 16 completed trials are summarized in Table 1. Currently, there has been no study conducted that has determined the role and/or outcomes of early initiation of these biologics in CRSwNP, which represents a future area of research. Thus, this white paper is meant to provide guidance in the use of biologic treatments in patients with upper airway disease.

## Methods

A national multidisciplinary expert panel of 34 certified specialists was created, composed of 16 rhinologists drawn from The Canadian Rhinology Working Group of the Canadian Society of Otolaryngology-Head & Neck Surgery, 7 allergists, and 11 respirologists. To facilitate expert panel selection, respirologists and allergists who were geographically diverse and with a demonstrated research interest in lower and upper airway diseases were identified and asked to participate. A systematic literature search for all randomized control trials involving CRSwNP and biologics was performed and disseminated to the group for review. The development of the recommendations were established through an adoption of the modified Delphi process [23].

The recommendation statements along with the corresponding supporting literature were compiled into a survey and provided to the expert panel with instructions and descriptions of how to complete the evaluation. Consistent with the modified Delphi model process, three rounds of anonymous independent recommendation statement survey ratings were conducted in which the first two rounds were completed individually online, and the third round was discussed on a virtual platform with all expert panelists. The Round 1 questionnaire consisted of 20 provided statements that were established by the lead author and are referred to as the ‘provided statements’ in the subsequent rounds. To reduce bias, all panelists were able to add new statement recommendations to the questionnaire to fill in knowledge gaps not covered by the provided statements. These new statement additions were referred to as ‘panelist statements’ in the subsequent rounds. To determine consensus in the first and second rounds, the “nine-point” scale was used with ratings of 1 to 9 (1–3 = Disagree; 4–6 = Neutral; 7–9 = Agree) for each recommendation. The statements, descriptive statistics and inter-rater reliability from all three rounds are shown in Tables 3, 4, 5 in "Appendix". For the third round, the “three-point” scale was used to determine consensus with ratings of 1 to 3 (1 = Disagree; 2 = Neutral; 3 = Agree) as recommended by Lange et al. [24]. The panelists were encouraged to provide commentary as they deemed necessary.

Ratings were quantitatively reviewed by mean, median, mode, range and standard deviation. Consensus was defined by relative reliability measures—kappa coefficient. According to the classification of Landis et al., kappa scores were interpreted as follows: $\kappa$ value < 0.00 indicated poor agreement, 0.00–0.20 slight, 0.20 to 0.40 moderate, 0.61 to 0.80 substantial and > 0.81 almost perfect agreement [25]. A $\kappa$ value > 0.61 was deemed appropriate for reliability. Statements that had overall ratings of 1 to 3 (disagree) with substantial agreement were removed for subsequent rounds. During the third round of the process, statements were discussed and re-analysed until inter-rater reliability $\kappa$ value of at least 0.61 (substantial agreement) was achieved.

Following three rounds of the modified Delphi process, the information was compiled, and recommendation statements that obtained full consensus with substantial agreement were selected for inclusion in this white paper.

## Results

Twenty recommendations were initially developed based on available evidence (Table 3 in "Appendix"). Following the first round of evaluations, the 20 recommendations were revised based on expert panel suggestions and re-distributed (Table 4 in "Appendix"). After the second round of evaluations, four recommendations were removed based on panelist ratings and high inter-rater reliability. The statements were further revised with the generation of new statements, and 35 recommendations were re-distributed to panelists for review before the third-round virtual conference (Table 5 in "Appendix"). The virtual conference was used to discuss clinical evidence behind controversial recommendations, their relevance, and ways of strengthening the wording of recommendations to gain greater panel acceptance. Following the third round of the modified Delphi process, 22 statements out of 35 were deemed appropriate with substantial agreement and were arranged according to patient population, biologic markers, biologic response, safety profile, and cost of biologics (Table 2).

**Table 2:** Consensus statements for use of biologics in upper airway disease

	Statement	Recommendation
*Patient Population*		
1	Patients with chronic symptoms of upper airway disease which include facial pressure/pain, nasal obstruction/congestion, nasal discharge or a loss of smell should be evaluated for upper airway disease	Recommendation
2	Patients treated appropriately for asthma with persistent chronic upper airway symptoms should be referred for further evaluation of upper airway disease	Recommendation
3	All CRSwNP patients with lower respiratory symptoms who have not previously been evaluated for asthma should be evaluated for possible asthma and referred to a clinician who can provide a systematic evaluation	Recommendation
4	Clinician(s) evaluating for upper airway disease should evaluate the nose with nasal endoscopy or in communities where no nasal endoscopy is available, anterior rhinoscopy is acceptable when the diagnosis of nasal polyps is apparent. If nasal endoscopy is unremarkable or unavailable, a CT scan could be ordered to rule out sinus disease without polyps	Recommendation
5	CT reports indicating polyps are not sufficient to make the diagnosis of CRSwNP and starting on biologics	Recommendation
6	All endotypes of CRSwNP confirmed by endoscopy or anterior rhinoscopy are considered eligible for a trial of biologic therapy	Recommendation
7	Biologics should be principally considered for those who have undergone adequate sinus surgery within the past 5 years and are refractory to oral and nasal steroids. Patients unsuitable for surgery who have failed medical therapy may also be considered candidates for biologic therapy based on shared patient decision making	Recommendation
8	The adequacy of previous surgery matters in determining if subsequent surgical management is required versus initiation of biologic therapy. This could be evaluated with a CT scan and/or endoscopy to determine if each of the diseased sinus cavities can receive appropriate topical drug delivery	Recommendation
9	Patients with refractory CRSwNP after surgery should be counselled regarding their options which include revision sinus surgery or biologics. Referral to a specialist that can counsel and/or perform extended surgical procedures should be sought if available	Recommendation
10	Patients with CRSwNP do not need co-existing Type 2 inflammatory condition such as asthma to be considered for biologic therapy	Recommendation
11	For most patients, CRSwNP symptoms need to be severe based on the clinician’s choice of a validated patient reported outcome measure (PROM) for chronic sinus disease to warrant the use of biologics. There are a subgroup of patients that may score lower than severe disease on a PROM due to acclimatization to their symptoms (i.e. allergic fungal rhinosinusitis and chronic prednisone users) and these cases should be considered for biologics based on shared decision making	Recommendation
12	In patients with CRSwNP and coexisting asthma, who qualify for a biologic therapy based on upper airway indications, a consultation with a specialist experienced in managing asthma is recommended before choosing the most appropriate biologic	Recommendation
13	There is insufficient evidence to make a recommendation for providing biologics to patients with CRSsNP	Recommendation
14	Where possible, patients with Aspirin Exacerbated Respiratory Disease (AERD) should be preferentially managed by a multidisciplinary team	Recommendation
*Biological Markers*		
15	At the time of writing, there are no biological markers required to start CRSwNP patients on biologics nor any markers to indicate best biologic to use	Option
*Biological Response*		
16	Nasal response to biologics should be assessed by 16 weeks after initiating biologic therapy with subjective and objective measures. If these improvements are not met at 16 weeks, the biologic should be re-evaluated	Recommendation
17	Patients should be evaluated every 6 months in the first two years of biologic initiation and yearly thereafter	Recommendation
19	When treating co-existing CRSwNP and asthma, an attempt should be made to obtain optimal results with a single biologic in both diseases	Recommendation
19	Pre-biologic criteria may be used to qualify a patient for a second or subsequent biologic therapies in case of sub-optimal response to the first biologic	Recommendation
20	CRSwNP who have exhausted biologics and not achieved simultaneous adequate response in both the upper and lower airways could be evaluated for possible revision sinus surgery	Recommendation
*Safety Profile*		
21	The risk of side effects is low in the short-term use of biologics in CRSwNP	Recommendation
*Cost of Biologics*		
22	Cost and access to biologics should be considered in the decision making of the use of biologics	Recommendation

In total, six recommendations did not reach consensus regarding their appropriateness. The statements that were removed throughout the modified Delphi process are not included here as this document only contains final agreed upon statements to provide the reader with clear statements regarding the use of biologics in upper airway diseases. Refer to Table 2 for a more comprehensive outline of each statement and the modified Delphi process.

## Discussion

### Consensus statements

After three rounds of the Modified Delphi process, 25 consensus statements were created and deemed appropriate for recommendations (Table 2).

#### Patient population

1. *Recommendation: Patients with chronic symptoms of upper airway disease which include facial pressure/pain, nasal obstruction/congestion, nasal discharge or a loss of smell should be evaluated for upper airway disease.*

CRS, an upper airway disease, is defined as sinonasal inflammation persisting for at least eight weeks. This definition is based on expert consensus and has been consistent across multiple CRS diagnosis and management guidelines in Canada, Europe and the United States [21, 26, 27]. Biologics have been largely studied in patients with CRSwNP [20]. Therefore, patients who have been diagnosed with CRSwNP, based on the current Canadian clinical practice guidelines (CPG) for CRSwNP, may be eligible for biologic treatment if both subjective and objective findings are observed. The symptom-based criteria for diagnosis CRSwNP is defined by having 2 or more of the following symptoms lasting at least eight weeks [20]:

- Facial congestion/fullness
- Facial pain/pressure
- Nasal obstruction/blockage
- Purulent anterior/posterior nasal drainage
- Hyposmia/anosmia

These symptoms must be accompanied by objective findings (see Statements 4 and 5) to meet eligibility for biologic therapy.

2. *Recommendation: Patients treated appropriately for asthma with persistent chronic upper airway symptoms should be referred for further evaluation of upper airway disease.*

CRSwNP and asthma frequently co-exist as manifestations of a common Type 2 inflammatory process within the contiguous upper and lower airways [1]. These diseases share several of the same histopathological changes, common inflammatory mediators, and the same primary effector cell (eosinophil) [28]. There is evidence that defects in the airway epithelial barrier function are associated with asthma and CRSwNP [29]. These defects in barrier function may play a critical role in the pathogenesis of CRSwNP by allowing an influx of foreign antigens into the submucosa where they may trigger or exacerbate an inflammatory response. The reported incidence of asthma varies from 2 to 66% in CRSwNP [30–34]. CRS has been postulated as a risk factor for the development of asthma and a biomarker of its severity.

Treatments for CRSwNP or asthma may improve the coexisting condition. When sub-optimally controlled, both CRSsNP and CRSwNP worsen the course of lower airway disease [35]. Patients may be receiving appropriate asthma therapy but if they have upper airway symptoms, these patients should be referred for evaluation of upper airway disease given both upper and lower airway disease frequently coexist together. Early management is imperative for improved quality of life and function [30, 36].

The Global Initiative for Asthma (GINA) 2022 annual report recommends the assessment of comorbidities including CRS as an important step in the global management of asthma [37]. As such, the expert panelists recommend clinicians screen asthma patients for upper airway disease.

3. *Recommendation: All CRSwNP patients with lower respiratory symptoms who have not previously been evaluated for asthma should be assessed for possible asthma and referred to a clinician who can provide a systematic evaluation.*

The prevalence of asthma in the Canadian population is reported at approximately 8.4% and increases to from 20 to 60% in CRSwNP patients [30-34, 38]. CRSwNP tends to be associated with adult-onset asthma (age greater than 18 years), and a subset are associated with late-onset asthma (age greater than 40 years); thus further highlighting the need to screen all CRSwNP patients for asthma [39, 40]. In the Global Allergy and Asthma European Network sinusitis cohort involving 52,000 subjects, approximately 50% of CRSwNP patients developed asthma [41]. Asthma has been identified as a premorbid condition for patients with CRS and is associated with a greater CRSwNP disease severity, higher recurrence rates, and reduced quality of life [42, 43]. Similarly, the presence of CRSwNP is associated with worse asthma outcomes including increased asthma symptoms, more asthma-related emergency department visits, hospitalizations, systemic corticosteroid use, and increased rates of revision surgery [19, 44, 45]. Thus, it is important for clinicians to be aware of the frequent coexistence of lower airway conditions in patients with CRSwNP as early identification and treatment can improve outcomes.

All clinician(s) who manage CRSwNP should evaluate patients for asthma by an appropriate history. Asthma history can be identified by asking the following questions, as described by the GINA report [46].

- Do you have a history of variable respiratory symptoms including wheeze, shortness of breath, chest tightness, and/or cough?
- Do your symptom(s) occur variably over time and in intensity?
- Do your symptom(s) often occur or are worse at night or on waking?
- Are your symptom(s) often triggered by exercise, laughter, allergens or cold air?
- Do your symptom(s) often occur with or worsen with viral infections?

Clinicians who are concerned about asthma should then refer the patient to clinician(s) who manage asthma. Comprehensive work up should include pulmonary function tests, blood work for serum IgE and eosinophils levels, allergy testing and, if available, measurement of exhaled nitric oxide (fractional exhaled nitric oxide or FeNO levels) [47]. Patient-reported questionnaires may be useful to assess asthma control and impact. For example, clinicians could consider the Asthma Control Questionnaire-5 or 6 (ACQ-5/6) or the Asthma Quality of Life Questionnaire (AQLQ) which were used in some CRSwNP randomized controlled trials (Table 1). The ACQ-5 or 6 are used to assess disease control and the AQLQ is used to assess quality of life of asthmatic patients, including the physical, occupational, emotional and social domains of patients.

4. *Recommendation: Clinician(s) evaluating for upper airway disease should evaluate the nose with nasal endoscopy or in communities where no nasal endoscopy is available, anterior rhinoscopy is acceptable when the diagnosis of nasal polyps is apparent. If nasal endoscopy is unremarkable or unavailable, a CT scan could be ordered to rule out sinus disease without polyps.*

When diagnosing CRSwNP, symptoms alone have a high sensitivity but a lower specificity, which is why both subjective and objective findings must be present to be eligible for biologic therapy [36, 48]. Endoscopy has high specificity and pre-test probability in confirming a CRSwNP diagnosis. Specialists must be cognisant that unilateral polyp disease can be caused by localized pathology such as fungal ball, antrochoanal polyps, odontogenic sinusitis or a tumour, either benign or malignant, and these diagnoses do not benefit from the use of biologic therapy [49].

In communities where nasal endoscopy is not readily accessible, anterior rhinoscopy may confirm diagnosis if frank bilateral polyposis is seen on examination. Anterior rhinoscopy, however, provides inconsistent visualization of structures past the inferior turbinate and therefore does not effectively rule out a diagnosis of nasal polyposis when normal [34]. Nasal endoscopy provides a more thorough examination of sinus drainage pathways in the middle meatuses, sphenoethmoidal recesses, and nasopharynx, and thus, anterior rhinoscopy should only be reserved for cases where nasal endoscopy is unavailable within the region.

Clinicians should obtain CT imaging in patients with symptoms of CRS and negative nasal endoscopy findings of polyps to rule out CRSsNP. Despite the high specificity and positive predictive value of nasal endoscopy in confirming the diagnosis of CRS, endoscopy is less sensitive than CT and thus has a high false-negative rate in ruling out patients with CRSsNP as nasal endoscopy cannot reliably assess for inflammation in surgically unopened sinus cavities. Given the high sensitivity of CT scanning, it can be used to rule out CRSsNP in this cohort of patients (in particular CRSsNP). From a cost-efficiency standpoint, obtaining a CT in a symptomatic patient with negative endoscopy findings is less costly due to savings from unnecessary future medical treatment and otolaryngologist visits [26].

5. *Recommendation: CT reports indicating polyps are not sufficient to make the diagnosis of CRSwNP and to initiate biologic therapy.*

CT scan reports may indicate polyp disease but these reports are unreliable given difficulty in differentiating between polyps and thick, inflamed mucosal changes, which often accompany upper respiratory tract infections and/or asymptomatic changes in the non-diseased population [50]. Given CT scans have a lower specificity than nasal endoscopy as described in Statements 4 and 5, this imaging technique is not sufficient to rule in or diagnose CRSwNP [51, 52]. Thus, to diagnose CRSwNP and initiate biologic therapy, the expert panel agrees that nasal endoscopy when available or anterior rhinoscopy where appropriate are the most reliable means of diagnosis.

6. *Recommendation: All endotypes of CRSwNP confirmed by endoscopy or anterior rhinoscopy are considered eligible for a trial of biologic therapy.*

In CRSwNP, biologic agents currently approved or under assessment for CRSwNP target components of the Type 2 inflammatory pathway [20]. There are several endotypes of CRSwNP defined by different pathogenic mechanisms. The current pathophysiological features of some asthma-related CRSwNP (allergic fungal rhinosinusitis and AERD) are well defined and regarded as known endotypes of CRSwNP involving the Type 2 inflammatory pathway. Eosinophilic granulomatosis with polyangiitis (EGPA), a rare multisystem disease characterized by asthma, CRSwNP, blood and tissue eosinophilia with vasculitis, is another condition where the pathophysiology is compatible with a Type 2 inflammatory mechanism. In severe EGPA cases, eosinophilic polyposis is recalcitrant to endoscopic sinus surgery (ESS) and intranasal corticosteroid spray (INCS) treatments, and these patients may benefit from initiation of biologic therapy [53]. In addition, IgE-mediated allergy has been a suggested cause of CRSwNP [54]. Allergy has always been strongly associated with a Type 2 inflammatory response (the underlying pathogenesis of CRSwNP). However, some diseases such as primary ciliary dyskinesia (PCD) and cystic fibrosis (CF) present with nasal polyps, but their endotype may not be driven by a Type 2 inflammatory mechanism.

Patients with PCD or CF are predisposed to CRS due to defective mucociliary clearance, which allows bacterial colonization of the sinuses [55]. Often, CRSwNP in PCD and CF is characterized by a neutrophilic histotype [56]. Despite primarily a Type 1 inflammatory mechanism, eosinophilic polyposis has been reported in both PCD and CF [55]. Although these patients were not included in the clinical trials, the expert panel agrees that biologic therapy may be considered in these patients on a case-by-case basis and in discussion with their primary PCF or CF physician.

7. *Recommendation: Biologics should be principally considered for those who have undergone adequate sinus surgery within the past 5 years and are refractory to oral and nasal steroids. Patients unsuitable for surgery who have failed medical therapy may also be considered candidates for biologic therapy based on shared patient decision making.*

A systematic review of 45 studies comprised of 34,220 patients by Loftus et al. demonstrated an overall revision rate of 18.6% in CRSwNP patients after ESS over eight years of follow-up [57]. In the review by Loftus et al., there were increased revision rates with more severe disease [57]. Both AERD and allergic fungal rhinosinusitis patients had higher revisions rates than the CRSwNP overall rate (27.2% and 28.7% vs. 18.6%, respectively) [57]. Hence, patients who develop recurrence after ESS are more likely to have severe disease and develop recurrences following subsequent revision ESSs. Thus, these patients have a greater risk of recalcitrant disease and should be considered for biologic therapy. Revision surgery is more appropriate for late polyp recurrence as a more cost-effective intervention than biologics as described by Scangas and colleagues [58]. Late recurrence implies that appropriate control can be achieved with surgery and standard medical therapy. Therefore, the expert panel defines early recurrence of nasal polyposis as development of polyps within 5 years after adequate ESS. Adequate ESS promotes ventilation, addresses mucostasis, and facilitates application of topical medical therapy, all essential goals of ESS [49].

Furthermore, patients who cannot undergo surgery due to medical comorbidities but fail appropriate medical therapy may benefit from biologic therapies, as they cannot receive the full benefits of topical medical therapy due to unopened paranasal sinuses.

8. *Recommendation: The adequacy of previous surgery matters in determining if subsequent surgical management is required versus initiation of biologic therapy. This could be evaluated with a CT scan and/or endoscopy to determine if each of the diseased sinus cavities can receive appropriate topical drug delivery.*

Adequate sinus surgery that promotes ventilation, addresses mucostasis, and facilitates application of topical medical therapy are essential goals in sinus surgery [49]. CRSwNP patients who have significant recurrence following ESS should be re-evaluated with endoscopy and CT scan to assess if adequate surgery was performed and whether further surgery is required [21]. If a patient is new to a surgeon, a CT scan should be obtained in addition to performing nasal endoscopy to evaluate the extent of previous surgery. If a patient is known to the surgeon, there should be documentation that openings to all diseased sinus cavities had been achieved prior to polyp recurrence. It is important to note that prior documentation may not address this or that operative notes may overstate the extent of sinus opening and thus, one must use clinical judgement to determine need for further evaluation with a CT scan and/or endoscopy. If there is no documentation, a CT scan should be obtained to ensure adequate surgery has been performed. Following this, the patient can then be considered for alternative therapies, such as biologics.

9. *Recommendation: Patients with refractory CRSwNP after surgery should be counselled regarding their options which include revision sinus surgery or biologics. Referral to a specialist that can counsel and/or perform extended surgical procedures should be sought if available.*

CRSwNP patients who suffer significant unresolved disease after apparently adequate ESS are often high-risk groups with AERD, asthma, and/or poorly controlled allergies. These cohorts of patients need to know their options which may include revision and extended surgical aeration approaches versus being placed on biologics. There are different degrees of extended sinus aeration approaches which are geared towards making each sinus cavity opening larger into a neo-sinus that has higher likelihood of remaining patent. A common extended sinus aeration approach is the endoscopic modified Lothrop procedure (Draf III) [59]. In patients with CRSwNP and comorbid asthma, Draf III approaches have been shown to yield lower revision surgery rates and longer time to disease recurrence post-surgery than patients receiving standard ESS [59]; however, this is still debated among many as symptomatic polyp recurrence can still occur. Understandably, geographical distance/remoteness and need for follow up may be barriers to referral to surgeons who perform such extended sinus procedures and patients may be started on a biologic as a result.

10. *Recommendation: Patients with CRSwNP do not need co-existing Type 2 inflammatory condition such as asthma to be considered for biologic therapy.*

Historically, prior to the approval of biologic therapies for CRSwNP, clinicians would prescribe biologics for patients suffering from asthma or atopic dermatitis and patients with CRSwNP indirectly benefit in this way. Both asthma and atopic dermatitis are Type 2 inflammatory diseases that currently have indications for the use of biologics in Canada. There is clear evidence that patients with CRSwNP, with or without other Type 2 inflammatory conditions, benefit from biologic therapy. For instance, the efficacy of dupilumab was investigated in patients with CRSwNP regardless of whether they had any other Type 2 mediated diseases [3]. Dupilumab is a human monoclonal antibody to interleukin 4 receptor α inhibiting IL-4 and IL-13, both of which both play a central role in Type 2 inflammation. In one of the clinical trials [3], there was no significant change in the primary endpoint of endoscopic nasal polyp score in patients without asthma treated with dupilumab. However, dupilumab did result in significant improvements in the secondary endpoints in this cohort of patients: total Sino-Nasal Outcome Test-22 (SNOT-22) scores, Lund-Mackay score on CT scan, and objective olfactory scores compared to the placebo group [3]. Those with comorbid asthma, representing a more severe disease Type 2 phenotype, aside from clinical improvement also had a significant improvement in nasal polyp score on dupilumab compared to placebo [3]. These results are similar to those of the other three randomized controlled trials that included an asthma cohort, but did not require asthma as a criterion to participate [3, 15, 60].

11. *Recommendation: For most patients, CRSwNP symptoms need to be severe based on the clinician’s choice of a validated patient reported outcome measure (PROM) for chronic sinus disease to warrant the use of biologics. There are a subgroup of patients that may score lower than severe disease on a PROM due to acclimatization to their symptoms (i.e. allergic fungal rhinosinusitis and chronic prednisone users) and these cases should be considered for biologics based on shared decision making.*

Examples of frequently used outcome measures for assessing subjective symptoms include, but are not limited to, the SNOT-22, Chronic Sinusitis Survey (CSS), and the Rhinosinusitis Disability Index (RSDI) for chronic rhinosinusitis symptoms [61]. In the randomized control trials that have been conducted with biologics targeting Type 2 inflammation in CRSwNP, most studies used the validated patient reported outcome, SNOT-22 (Table 1). Otherwise, a Visual Analogue Scale (VAS) score, which is not a validated PROM, was also used in combination with the SNOT-22 or on its own. Other controlled trials commonly used another non-validated “total symptom score” with a scale range of 0 to 9 points.

Patients require severe symptoms based on the Health Canada recommendation to be eligible for biologic treatment. For example, a SNOT-22 score of > 50 is considered severe CRSwNP disease [62].

Clinicians should be cautious when interpreting PROM scores as PROMs are subject to change from biases inherent to self-reporting, often referred to as a “response shift” [63]. There are clinical scenarios involving sinus pathology with minimally affected PROM scores due to patients’ acclimatization to their symptoms. For example, allergic fungal rhinosinusitis and chronic prednisone users typically have normal to minimally affected SNOT-22 scores, but these patients still require medical and/or surgical intervention to correct the underlying disease process [64]. Although these patients may not be stratified as “severe” based on their PROM scores, they should still be considered for further management options for their CRSwNP disease, including biologic therapy.

12. *Recommendation: In patients with CRSwNP and coexisting asthma who qualify for a biologic therapy based on upper airway indications, a consultation with a specialist experienced in managing asthma is recommended before choosing the most appropriate biologic.*

Given biologics target specific inflammatory markers involved in the pathophysiology of CRSwNP, patients suffering from coexisting CRSwNP and asthma may derive a further benefit from biologics. All biologics currently approved for CRSwNP are also approved for use in asthma. However, response to biologic therapy in asthma have been shown to be dependent on several clinical features and biomarkers. Before starting a biologic therapy for CRSwNP with comorbid severe asthma, appropriate steps should be taken to assess if such therapy is also required for asthma, and which biologic agent is the most appropriate to adequately target both diseases. A preliminary study conducted on patients with recalcitrant asthma and CRSwNP showed that biologics were beneficial for both airway diseases [65].

13. *Recommendation: There is insufficient evidence to make a recommendation for providing biologics to patients with CRSsNP.*

Currently, there are no published studies which investigated the use of biologics in CRSsNP for the panel to consider. CRSsNP has not been studied, but the diversity of inflammatory profiles in CRSsNP suggests Type 2 inflammation may play a role in a subset of patients and trials are currently underway to assess the efficacy of this therapy. However, CRSsNP patients with comorbid asthma may be treated with biologic therapy based on their comorbid disease indication.

14. *Recommendation: Where possible, patients with Aspirin Exacerbated Respiratory Disease (AERD) should be preferentially managed by a multidisciplinary team.*

AERD is characterized by CRSwNP, asthma, and distinct respiratory reactions to aspirin and other non-specific nonsteroidal anti-inflammatory drugs (NSAIDs) [66]. The prevalence of AERD among CRSwNP patients is approximately 10%, and generally, these are amongst the most difficult to treat CRSwNP patients due to the severity of the underlying inflammation, leading to disease recalcitrance [66–68]. This is reflected at the cellular and molecular level; nasal polyps from patients with AERD have been shown to have over three times as many eosinophils and higher IL-5 concentrations when compared to polyps from subjects with non-AERD CRS [69, 70]. This tends to correlate with an increased risk of postoperative polyp disease recurrence [71].

Given the complexity of this disease, AERD patients should be managed by a multidisciplinary team. For conservative management, these patients should receive appropriate medical therapy for both their asthma and CRSwNP diseases. AERD patients who remain refractory to medical management should be considered for surgical intervention. ESS is the mainstay treatment for nasal polyp removal with significant improvements in endoscopic, radiographic and subjective measures in this cohort of patients [72–75]. However, the durability of benefit is generally shorter than for non-AERD CRS patients and thus, these patients more often require revision surgeries due to disease recurrence [76]. Among patients with CRSwNP alone, CRSwNP with asthma, and CRSwNP and AERD, median time to polyp recurrence were 20, 4, and 0.66 years, respectively [77]. Furthermore, a systematic review of 45 studies showed revisions rates in CRSwNP patients with asthma (22.6%), AERD (27.2%), and allergic fungal rhinosinusitis (28.5%) had higher revision rates in comparison to patients with CRSwNP alone (22.6%) [57]. If possible, surgeons managing AERD patients should be comfortable performing advanced aeration surgical procedures. Advanced aeration surgery such as Draf III has, in the setting of AERD, have been shown to have positive outcomes including greater quality of life, improved disease maintenance, and reduced polyp recurrence [78, 79]. There is additional data that has emerged that complete sinus surgery followed by aspirin desensitization and long-term aspirin maintenance leads to long-term symptom disease control [80, 81].

CRSwNP symptoms for many AERD patients may be refractory after surgery with concurrent medical management, and biologics should be considered in this patient cohort for management of both their asthma and CRSwNP diseases if they are eligible [3, 4]. The included randomized controlled trials have demonstrated significant improvements in nasal polyp scores, CT imaging, morning nasal congestion and obstruction scores, and sense of smell (Table 1). This should be a shared decision between the patient and clinician as the stakes of the treatment decisions in AERD are high. The risks and benefits of further surgical intervention and long-term injectable medication must be considered, while also considering patient resources. Recently, dedicated cost-effectiveness and health utility studies have begun to address biologics in AERD. In a study by Yong et al., biologics were found to be cost-effective as salvage therapy after aspirin desensitization for treatment of AERD and biologic use resulted in fewer ESS revision surgeries than appropriate medical management and aspirin desensitization after ESS [82]. However, a recent systematic review and meta-analysis by Chu et al. showed that although aspirin desensitization can improve AERD quality of life and upper airway symptoms, these benefits are counterbalanced by an increased risk of adverse events [83]. Common side effects of aspirin desensitization include major bleeding, gastritis, asthma exacerbation and rashes, which often result in treatment discontinuation in this cohort of patients [83]. It is also important to note that aspirin desensitization is not widely available across Canada, which presents as another barrier for this cohort of patients in accessing and utilizing treatments for AERD. Thus, clinicians must undertake an individualized, patient-centered care approach to managing AERD patients, considering the availability and the risks and benefits of aspirin desensitization and possible treatment alternatives which include biologic therapy.

Multidisciplinary evaluation of AERD patients is important before deciding upon upper airway treatment as many patients may qualify for biologic therapy for their asthma. If such treatment is deemed necessary for the asthma component of the triad, given the concomitant efficacy on comorbid CRSwNP, it would be advised to delay further treatment decisions concerning upper airway disease until residual disease on biologic therapy has been assessed [84].

#### Biologic markers

15. *Option: At the time of writing, there are no biological markers required to start CRSwNP patients on biologics nor any markers to indicate best biologic to use.*

The inclusion criteria of all randomized control trials evaluating the efficacy of biologics in CRSwNP patients used clinical findings and no biological markers; therefore, no recommendations can be made regarding biological markers required to start, evaluate therapeutic response, nor predict the best biologic to use for an individual.

#### Biologic response

16. *Recommendation: Nasal response to biologics should be assessed between 16 weeks after initiating biologic therapy with subjective and objective measures. If these improvements are not met after 16 weeks, the biologic should be re-evaluated.*

The definition of response is complex but requires subjective and objective improvement within a defined time frame. Based on clinical trial data, 16 weeks appears to provide sufficient time to determine if the biologic therapy had a positive impact on subjective and objective outcomes in patients with CRSwNP. The expert panel agrees there must be a discussion between the clinician and the patient to determine if the improvements achieved merit continuing biologic therapy at 16 weeks follow-up.

For subjective measures, the clinician may use the patient reported outcome measures (PROMs) used initially to define severity of symptoms to compare if there was a minimal clinically important difference (MCID) in subjective symptoms by 16 weeks. It is important to understand that changes in PROM scores that are statistically significant may not correlate with meaningful changes in patient experience [85]. The validated PROMs most frequently used in the randomized controlled trials included the SNOT-22, RSDI, CSS, The University of Pennsylvania Smell Identification Test (UPSIT), or Sniffin’ Sticks Test (Table 1). To assess the lower airways, the PROMs most commonly used in the trials include the 6-question Asthma Control Questionnaire (ACQ-6) and the 5-question Asthma Control ﻿Questionnaire (ACQ-5). Other validated questionnaires include the 31-item Rhinosinusitis Outcome Measure (RSOM-31) for assessment of rhinosinusitis outcomes and the 36-Item Short Form Survey (SF-36) and Generic health-related quality of life questionnaire (EQ-5D) for assessment of overall health-related quality of life. See below for specific MCIDs of various PROMs used to assess CRSwNP and asthma.

For objective measures, the committee supports the use of endoscopy over CT scan. Clinicians are recommended to use a validated endoscopy grading rubric to help compare endoscopy findings before and after 16 weeks of treatment. There is a limitation of polyp grading scales where there is a significant reduction in the size of the polyp and symptomatic improvement despite the polyp grade not improving with treatment; therefore, subjective improvements are considered in conjunction to determine efficacy of the biologic.

The MCID of the following PROMs commonly used in symptom assessment of CRSwNP:

- SNOT-22: MCID = 8.9, total score range = 0 to 120 where higher scores indicate greater impact of disease [86, 87]
- CSS: MCID = 9.75, total score range = 0 to 100 where lower scores indicate greater impact of disease [88]
- RSDI: MCID = 10.35, total score range = 0 to 120 where higher scores indicate greater impact of disease [89]

In all clinical trials, the minimum score improvement observed at 16 weeks on biologic therapy for the SNOT-22 was greater than the MCID with a minimum score improvement of 15.

The lower airway is frequently assessed with the ACQ-5 or 6 and AQLQ validated questionnaires. The MCID of the following PROMs used in symptom assessment for asthma in the clinical trials included:

- ACQ-5 or 6: 0.5 [90–92]
- AQLQ: 0.5 [93–96]

Although CRSwNP trials demonstrated an improvement greater than the MCID in asthma PROMs for patients with comorbid asthma, asthma clinical trials have not shown such a consistent improvement in PROMs when compared to placebo. The expected benefit and assessment of response in asthma is primarily the reduction in exacerbation and/or oral corticosteroid (OCS) dose in OCS-dependant patients.

17. *Recommended: Patients should be evaluated every 6 months in the first two years of biologic initiation and yearly thereafter.*

It is important to monitor the patient’s response to a biologic drug once it has been selected to treat the upper or lower airways. Non-responders may be expected in 25% to 50% of cases depending on the biologic chosen and the outcome being measured [97]. To avoid inadequate treatment and associated unnecessary costs to the patient and healthcare system, an expected response to the treatment should be reached within 4 to 6 months. Thus, patients should be evaluated every 6 months in the first two years of biologic initiation to ensure patient safety and appropriate use of healthcare resources. If the patient remains to be adequately controlled on biologic therapy after two years, clinicians may evaluate patients once annually. It is important that clinicians screen for adverse events related to biologic therapy at each visit (see Statement 24).

18. *Recommendation: When treating co-existing CRSwNP and asthma, an attempt should be made to obtain optimal results with a single biologic in both diseases.*

At this time, there are no guidelines regarding dual or combination biologic therapy in patients with upper and lower airway disease. Thus, clinicians should attempt to manage coexisting upper and lower airway diseases with one biologic.

However, the committee acknowledges that the landscape of biologics in upper and lower airway disease is constantly and quickly evolving with new evidence emerging for dual biologic use. There have been select case reports and case series which have investigated the use of dual biologic therapy in specific patients (i.e. patients with evidence of both allergic and eosinophilic inflammation) [98]. In a series of case reports, patients with severe asthma and comorbid disease (i.e. atopic dermatitis, CRSwNP, and AERD) who remained refractory despite maximal controller therapy, systemic steroids, and biologic monotherapy demonstrated marked improvement in symptom control, reduced asthma exacerbations, and reduced steroid use after the addition of a second biologic from a different class [98, 99]. Combinations of biologics from different classes were determined by treatable traits and included omalizumab and dupilumab, mepolizumab and omalizumab, and benralizumab and omalizumab [98].

19. *Recommendation: Pre-biologic criteria may be used to qualify a patient for a second or subsequent biologic therapies in case of sub-optimal response to the first biologic.*

There are three biologics approved for use in Canada for CRSwNP as of October 2022 and there are no randomized control studies that investigate outcomes following a switch in biologic therapy if a patient fails to improve or have residual impairment with their first prescribed biologic agent. In the case of significant residual impairment on biologic therapy, a switch in treatment may provide further benefits. But, as the first biologic may have improved some PROMs or objective measures or altered biomarkers, the committee recommends clinicians consider pre-biologic criteria when deciding on a second or subsequent biologic therapy until more evidence emerges on biologic switching.

It has been postulated that patients may derive a benefit from a biologic with a different target. This is common practice in asthma although the supporting evidence is limited. New data on biologic switching from anti-IgE to anti-eosinophil agents in dual-eligible asthmatic patients who did not respond to omalizumab have been published [100]. A multicentre clinical trial (OSMO) demonstrated switching to an anti-eosinophil biologic in asthmatic patients was safe and efficacious in improving asthma control, healthcare utilization and exacerbations, even without an omalizumab washout period [50]. In several other case series, patients with severe allergic asthma demonstrated improved symptom control and a reduction in asthma exacerbations and severity after switching from omalizumab to mepolizumab [101]. As biologics target different inflammatory receptors and cytokines, patients with a suboptimal response to omalizumab might benefit from an anti-eosinophil agent, depending on their treatable traits. However, the data supporting this continue to be limited and further research is needed to determine optimization via biologic switching between classes.

In addition, patients may benefit from a different biologic within the same pathway in patient-specific situations. It has been reported that some asthmatic patients with a more severe baseline disease, as measured by the ACQ-5/6, are less likely to respond to anti-IL-5 agents [102, 103]. Patients with more severe asthma likely have multiple treatable traits beyond eosinophilic inflammation driving their inflammation and resultant poor symptom control [102, 104]. In such circumstances, clinicians can consider switching biologics within the same pathway. Several retrospective reports have shown that the switch from mepolizumab in non-responders to benralizumab resulted in improvements in exacerbations, oral corticosteroid dose, and asthma control [105, 106]. Similar trends were demonstrated for switching from mepolizumab to reslizumab in a small single-blinded placebo-controlled trial [105]. However, despite these emerging findings, these observations are from studies with small sample sizes and more robust, prospective data is required to help inform biologic switching.

20. *Recommendation: CRSwNP patients who have exhausted biologics and not achieved simultaneous adequate response in both the upper and lower airways could be evaluated for possible revision sinus surgery.*

In this consensus, biologics are recommended for patients who have failed appropriate medical and surgical management. When CRSwNP patients remain refractory on biologic therapy, we recommend these patients be re-evaluated for revision sinus surgery. Surgery can be a cost-effective way to remove the recalcitrant polyps and to optimize medical management, which will have an indirect benefit for the lungs as it decreases the inflammatory load [107].

#### Safety of biologics

21. *Recommendation: The risk of side effects is low with short-term use of biologics in CRSwNP.*

At this time, there is evidence from published randomized controlled trials that the use of biologics in CRSwNP is considered safe for short-term use up to 52 weeks. The most common mild adverse events reported include headache, nasopharyngitis, upper respiratory tract infection, and oropharyngeal pain [108, 109]. Continuation of biologics in patients who develop mild side effects should be a shared-decision making process between the patient and clinician. Hypersensitivity reactions such as conjunctivitis, angioedema, hypotension, bronchospasm, urticaria and rashes may develop within hours of administration, but may have a delayed onset over days [110]. If a hypersensitivity reaction occurs, discontinuation of the biologic should be immediate with appropriate treatment for the hypersensitive reaction (110).

In regards to more severe, yet rare, events, recent data have shown that dupilumab can be associated with a transient increase in blood eosinophils and rare cases of eosinophilic pneumonia [111]. In these cases, patients commonly present with progressively worsening lower respiratory symptoms and there should be a low threshold for obtaining additional chest imaging to evaluate for pneumonia. It is however, not recommended to systematically assess this side effect if the patient remains asymptomatic. If patients develop these severe adverse reactions, the biologic should be discontinued. At this time, the expert panel cannot make recommendations on biologic switching in patients who develop severe adverse reactions due to lack of evidence as described in Statement 19. For guidance on initiation of a second or subsequent biologic, please reference Statement 19.

The safety of biologics for other indications such as asthma and atopic dermatitis have been researched more widely and demonstrate that they are safe for long-term use over years of use, and millions of injections [112, 113].

A number of contraindications to biologics have been discussed in the literature, some of which are listed below. However, there is insufficient data to determine absolute contraindications to biologics in pregnancy, breast-feeding, and helminth infections [114, 115]. Clinicians should be aware of absolute contraindications to biologics which include hamster protein hypersensitivity as these agents are produced in Chinese hamster ovary cells [116]. Overall, the contraindications to biologics are few and it is considered a relatively safe therapeutic option.

### Cost of biologics

22. *Recommendation: Cost and access to biologics should be considered in the decision making of the use of biologics.*

In a single payer health care system supported by private pharmaceutical insurance coverage, the cost of biologic therapy should be considered. Surgery remains a cost-effective option for most cases of CRSwNP. Generally, biologics in Canada indicated for asthma can range between CAD$600 to $4000 per vial/syringe, dependent on the drug [117, 118]. As the annual cost of biologics are high, their use should be restricted to appropriate cases where other options have been exhausted. Several cost utility analyses have shown that upfront surgery for CRSwNP is a more cost-effective option than a biologic [58], as such, ESS remains the most cost-effective treatment option and should be considered standard of care in CRSwNP patients refractory to medical therapy [119]. However, while important to note the importance of a complete, ‘full house’ ESS, it is evident that those who require revision surgery more than once may require it again and the time between surgeries often diminishes with each surgery. Therefore, clinicians must determine where there are diminishing returns with surgery and when best to proceed with biologic therapy and this white paper provides guidance in that decision algorithm.

## Conclusion

Management options for patients with CRSwNP includes the use of biologic therapies. While biologics have been used for several years in other conditions characterised by Type 2 inflammation, such as asthma and atopic dermatitis, they have recently emerged for the management of CRSwNP. This white paper provides guidance for appropriate use of biologics for upper airway disease through the lens of multidisciplinary specialists—rhinologists, allergists and respirologists. We expect this white paper to evolve over time and will require updating as additional clinical trials become available and clinical experience increases.

## Data Availability

The datasets used and/or analysed during the current study are available from the corresponding author on reasonable request.

## Appendix

See Tables 3, 4 and 5.

**Table 3:** Round 1 of the modified Delphi process for the consensus statements for use of biologics in upper airway disease

	Statement	Descriptive statistics	Inter-rater reliability	Decision
1	Patients with symptoms of upper airway disease which include facial pressure/pain, nasal obstruction/congestion, discharge or a loss of smell or severe/uncontrolled asthma should be evaluated for upper airway disease. Clinician(s) evaluating for upper airway disease should evaluate the nose with endoscopy or a CT sinus scan	Mean = 7.63 (Agree), Median = 8, Mode = 8 Total voters = 32	Fleiss’ Kappa = 0.617 (Substantial agreement)	Revised and re-assessed in Round 2 of the Modified Delphi Process
2	All endotypes of CRSwNP confirmed by endoscopy or anterior rhinoscopy are considered eligible except for primary ciliary dyskinesia and cystic fibrosis. CT reports indicating polyps is not sufficient in making the diagnosis of CRSwNP	Mean = 7.35 (Agree), Median = 8, Mode = 8 Total voters = 31	Fleiss Kappa = 0.598 (Moderate agreement)	Revised and re-assessed in Round 2 of the Modified Delphi Process
3	Biologics should only be considered for those who have undergone adequate sinus surgery and failed appropriate medical therapy (AMT) following surgery. Patients unfit for surgery who have failed AMT may also be considered candidates for biologic therapy. The adequacy of previous surgery matters in determining subsequent surgical management versus initiation of biologic therapy. Prior to initiation of biologics, there needs to be documentation on endoscopy and/or CT scan that all the partitions preventing adequate medical delivery to the sinuses had been achieved prior to polyp recurrence	Mean = 5.87 (Neutral), Median = 6, Mode = 9 Total voters = 31	Fleiss Kappa = 0.539 (Moderate agreement)	Revised and re-assessed in Round 2 of the Modified Delphi Process
4	Patients with CRSwNP do not need co-existing Type 2 inflammatory condition such as asthma to be considered for biologic therapy	Mean = 8.38 (Agree), Median = 9, Mode = 9 Total voters = 32	Fleiss Kappa = 0.772 (Substantial agreement)	Agreed upon; No vote required in Round 2 Modified Delphi Process
5	The severity of subjective CRSwNP symptoms needs to be moderate to severe based on the clinicians choosing of a validated patient reported outcome measure (PROM) for chronic sinus disease to warrant the use of biologics	Mean = 7.53 (Agree), Median = 8, Mode = 9 Total voters = 32	Fleiss Kappa = 0.584 (Moderate agreement)	Revised and re-assessed in Round 2 of the Modified Delphi Process
6	All CRSwNP patients should be evaluated for possible asthma. Patients with possible lower airway disease based on history should be referred to clinician(s) that can provide a systematic evaluation for asthma and allergy. Patients should be referred preferentially to clinicians who can organize pulmonary function tests, blood work for IgE and eosinophils levels, perform skin prick test and pulmonary FeNO levels as well as conduct a validated disease specific questionnaire for asthma	Mean = 6.84 (Neutral), Median = 7, Mode = 8 Total voters = 32	Fleiss Kappa = 0.561 (Moderate agreement)	Revised and re-assessed in Round 2 of the Modified Delphi Process
7	There is insufficient evidence to make a recommendation for providing biologics to patients with CRSsNP without asthma	Mean = 7.38 (Agree), Median = 8, Mode = 8 Total voters = 32	Fleiss Kappa = 0.583 (Moderate agreement)	Revised and re-assessed in Round 2 of the Modified Delphi Process
8	Patients with severe uncontrolled asthma or any other severe type 2 conditions in the setting of CRSsNP can be considered for biologics use based on their respective Canadian guidelines	Mean = 8.09 (Agree), Median = 8, Mode = 9 Total voters = 32	Fleiss Kappa = 0.640 (Substantial agreement)	Revised and re-assessed in Round 2 of the Modified Delphi Process
9	Biologics should not be provided to those suffering with recurrent acute bacterial sinusitis	Mean = 8.52 (Agree), Median = 9, Mode = 9 Total voters = 31	Fleiss Kappa = 0.749 (Substantial agreement)	Revised and re-assessed in Round 2 of the Modified Delphi Process
10	Where possible, if adequate surgery has been performed on CRSwNP patient, the patient should be assessed by an individual who can perform extended surgical approaches for further aeration of the sinuses. This surgeon can then determine if the patient would benefit from surgery or should proceed to starting a biologic	Mean = 5.74 (Neutral), Median = 6, Mode = 8 Total voters = 31	Fleiss Kappa = 0.537 (Moderate agreement)	Revised and re-assessed in Round 2 of the Modified Delphi Process
11	Patients with Aspirin Exacerbated Respiratory Disease (AERD) should be preferentially managed in centres with specialists who can perform advanced aeration surgeries for CRSwNP, manage severe/uncontrolled asthma, and provide possible aspirin desensitization	Mean = 6.42 (Agree), Median = 7, Mode = 8 Total voters = 31	Fleiss Kappa = 0.541 (Moderate agreement)	Revised and re-assessed in Round 2 of the Modified Delphi Process
12	Patients with CRSwNP who must wait longer than the provincial benchmark for undergoing sinus surgery should be allowed to initiate biologic therapy as a bridge to surgical management. Biologics are discontinued following surgery and only re-started if fits appropriate criteria based on these guidelines	Mean = 5.26 (Neutral), Median = 5, Mode = 7 Total voters = 31	Fleiss Kappa = 0.546 (Moderate agreement)	Revised and re-assessed in Round 2 of the Modified Delphi Process
13	Biologics can be uniquely considered for hyposmic patients where their sense of smell function is required for safety reasons or for their job if they have a history CRSwNP treated with surgery with adequate control of their disease and no evidence of polyps on endoscopy. Objective testing must be performed (UPSIT $\le 33$ or Sniffin’ Sticks Test $\le$ 30)	Mean = 6.0 (Neutral), Median = 6, Mode = 5 Total voters = 31	Fleiss Kappa = 0.535 (Moderate agreement)	Revised and re-assessed in Round 2 of the Modified Delphi Process
14	At the time of writing, there are no biological markers required to start CRSwNP patients on biologics nor any markers to indicate best biologic to use	Mean = 7.13 (Agree), Median = 7.5, Mode = 7 Total voters = 32	Fleiss Kappa = 0.583 (Moderate agreement)	Revised and re-assessed in Round 2 of the Modified Delphi Process
15	Response to biologics is based on subjective and objective improvement by 16 weeks. Patients should experience an improvement to some or all of their major upper airway symptoms which include sense of smell, nasal obstruction, nasal discharge, and facial pain. Furthermore, there should also be objective improvement on endoscopy or CT scan by 16 weeks and this should be re-evaluated at 1 year	Mean = 7.0 (Agree), Median = 7, Mode = 8 Total voters = 32	Fleiss Kappa = 0.595 (Moderate agreement)	Revised and re-assessed in Round 2 of the Modified Delphi Process
16	Clinicians using biologics to manage the both upper and lower airway disease should achieve MCID in both upper and lower airway validated questionnaires. If not, clinicians should discuss with one another if another biologic would benefit both the upper and lower airways better	Mean = 6.50 (Neutral), Median = 7, Mode = 7 Total voters = 32	Fleiss Kappa = 0.589 (Moderate agreement)	Revised and re-assessed in Round 2 of the Modified Delphi Process
17	Providers have the option of providing another biologic therapy if patients fail to respond to one biologic agent but continue to fit the inclusion criteria for biologic therapy. At this time, there are no biological markers to determine the best biological agent to use. There is no current literature to advise biological switching	Mean = 7.03 (Agree), Median = 7, Mode = 7 Total voters = 32	Fleiss Kappa = 0.612 (Substantial agreement)	Revised and re-assessed in Round 2 of the Modified Delphi Process
18	CRSwNP and asthma who have exhausted biologic switching and not achieved adequate response in either the upper or lower airways may consider dual biologic therapy. Dual biologic response should be evaluated at 16 weeks. If there are additional subjective/objective improvements, dual biologic therapy may be continued and re-evaluated at 1 year	Mean = 5.94 (Neutral), Median = 6, Mode = 8 Total voters = 32	Fleiss Kappa = 0.546 (Moderate agreement)	Revised and re-assessed in Round 2 of the Modified Delphi Process
19	The short-term use of biologics (12 months) in CRSwNP is considered safe. In other Type 2 inflammatory conditions, biologics have been shown to be safe long term	Mean = 7.06 (Agree), Median = 7.5, Mode = 8 Total voters = 32	Fleiss Kappa = 0.563 (Moderate agreement)	Revised and re-assessed in Round 2 of the Modified Delphi Process
20	Cost and access to biologics matters in the decision making of the use of biologics for CRSwNP patients with or without another Type 2 inflammatory condition	Mean = 7.23 (Agree), Median = 8, Mode = 9 Total voters = 32	Fleiss Kappa = 0.594 (Moderate agreement)	Revised and re-assessed in Round 2 of the Modified Delphi Process

**Table 4:** Round 2 of the modified Delphi process for the consensus statements for use of biologics in upper airway disease

	Statement	Descriptive Statistics	Inter-rater Reliability	Decision
1	Patients with symptoms of upper airway disease which include facial pressure/pain, nasal obstruction/congestion, nasal discharge or a loss of smell or uncontrolled asthma with persistent symptoms despite therapy should be evaluated for upper airway disease. Clinician(s) evaluating for upper airway disease should evaluate the nose with nasal endoscopy or in communities where no nasal endoscopy is available, anterior rhinoscopy is acceptable. If exam is unremarkable, a CT scan can be ordered to rule out sinus disease without polyps	Mean = 7 (Agree), Median = 8, Mode = 8 Total voters = 33	Fleiss’ Kappa = 0.595 (Moderate Agreement)	Revised and re-assessed in Round 3 of the Modified Delphi Process
2	All endotypes of CRSwNP confirmed by endoscopy or anterior rhinoscopy with a Nasal Polyp Score (NPS) of 5 and a biopsy confirming type 2 disease (eosinophilia > 10 HPF) are considered eligible for biologic therapy except for patients with antrochoanal polyp, primary ciliary dyskinesia, cystic fibrosis and vasculitis. CT reports indicating polyps are not sufficient to make the diagnosis of CRSwNP and/or starting on biologics	Mean = 5.84 (Neutral), Median = 7, Mode = 8 Total voters = 33	Fleiss’ Kappa = 0.54 (Moderate agreement)	Revised and re-assessed in Round 3 of the Modified Delphi Process
3	Biologics should be principally considered for those who have undergone adequate sinus surgery within the past 5 years and failed even with compliant use of nasal steroids and saline irrigation. Patients unfit for surgery who have failed medical therapy may also be considered candidates for biologic therapy based on shared patient decision making. The adequacy of previous surgery matters in determining if subsequent surgical management is required versus initiation of biologic therapy. Adequate surgery should be evaluated with a CT scan to determine if each of the sinus cavities can receive appropriate drug delivery	Mean = 6.50 (Neutral), Median = 7.5, Mode = 8 Total voters = 33	Fleiss’ Kappa = 0.558 (Moderate agreement)	Revised and re-assessed in Round 3 of the Modified Delphi Process
4	Statement 4 no vote			
5	The severity of subjective CRSwNP symptoms needs to be moderate to severe based on the clinician’s choice of a validated patient reported outcome measure (PROM) for chronic sinus disease to warrant the use of biologics. There are a subgroup of patients that may score lower than moderate disease on a PROM due to acclimatization to their symptoms (i.e. allergic fungal rhinosinusitis and chronic prednisone users) and these cases should be considered for biologics on a case-by-case basis	Mean = 7.28 (Agree), Median = 8, Mode = 8 Total voters = 33	Fleiss’ Kappa = 0.631 (Substantial agreement)	Revised and re-assessed in Round 3 of the Modified Delphi Process
6	All CRSwNP patients with lower respiratory symptoms who have not previously been evaluated for asthma should be evaluated for possible asthma and referred to a clinician who can provide a systematic evaluation. In a patient with CRSwNP qualifying for biologic therapy and severe asthma, a consultation with a specialist who can manage severe asthma is recommended before choosing the most appropriate biologic	Mean = 7.65 (Agree), Median = 8, Mode = 8 Total voters = 33	Fleiss’ Kappa = 0.601 (Substantial Agreement)	Revised and re-assessed in Round 3 of the Modified Delphi Process
7	There is insufficient evidence to make a recommendation for providing biologics to patients with CRSsNP	Mean = 7.31 (Agree), Median = 8, Mode = 8 Total voters = 33	Fleiss’ Kappa = 0.63 (Substantial agreement)	Agreed upon; No vote required in Round 3 Modified Delphi Process
8	Patients with severe uncontrolled asthma or any other severe type 2 conditions in the setting of CRSsNP can be considered for biologics use outside of clinical research trials, for those conditions other than CRSsNP, if they meet eligibility criteria for biologic therapy for another severe type 2 condition based on their respective Canadian guidelines	Mean = 6.52 (Neutral), Median = 7, Mode = 7 Total voters = 33	Fleiss’ Kappa = 0.531 (Moderate Agreement)	Revised and re-assessed in Round 3 of the Modified Delphi Process
9	Biologics should not be provided to those with recurrent acute bacterial sinusitis without CRSwNP	25 REMOVE: 8 KEEP (75.76%)		Removed based on majority vote
10	Where possible and there is no extended delay in assessment, if adequate surgery has been performed for a CRSwNP patient and the patient remains refractory, the patient should be evaluated by an individual who can perform extended surgical approaches. This is done in order to provide the patient comprehensive understanding of their options which include standard revision sinus surgery, extended surgical approaches or biologics	Mean = 5.78 (Neutral), Median = 6.5 Mode = 7 Total voters = 33	Fleiss’ Kappa = 0.507 (Moderate agreement)	Revised and re-assessed in Round 3 of the Modified Delphi Process
11	Where possible, patients with Aspirin Exacerbated Respiratory Disease (AERD) should be preferentially managed in centres that can provide all aspects of care: advanced aeration surgery, severe asthma management, aspirin desensitization and biologic treatment in order to provide options to the patient and optimize multi-modal care for these complex patients	Mean = 6.78 (Neutral), Median = 8, Mode = 8 Total voters = 33	Fleiss’ Kappa = 0.518 (Moderate agreement)	Revised and re-assessed in Round 3 of the Modified Delphi Process Revised and re-assessed in Round 3 of the Modified Delphi Process
12	Patients with CRSwNP who wait longer than 6 months for undergoing primary sinus surgery should be allowed to initiate biologic therapy as a bridge to surgical management. If a patient achieves desired symptom control on biologics prior to surgery, a patient may choose not to do surgery and continue with biologics	Mean = 6.48 (Neutral), Median = 7, Mode = 8 Total voters = 32	Fleiss Kappa = 0.535 (Moderate agreement)	
13	Biologics can be uniquely considered for hyposmic patients where their sense of smell function is required for safety reasons or for their job if they have a history CRSwNP treated with surgery with adequate control of their disease and no evidence of polyps on endoscopy. Objective testing must be performed (UPSIT $\le 33$ or Sniffin’ Sticks Test $\le$ 30)	24 REMOVE: 9 KEEP (72.72%)		Removed based on majority vote
14	At the time of writing, there are no biological markers required to start CRSwNP patients on biologics nor any markers to indicate best biologic to use	Mean = 7.13 (Agree), Median = 7.5, Mode = 7 Total voters = 32 18 REMOVE: 13 KEEP (56.25%)	Fleiss Kappa = 0.583 (Moderate agreement)	Revised and re-assessed in Round 3 of the Modified Delphi Process
15	Response to biologics is based on subjective improvement by 24 weeks. Patients should experience an improvement to some or all of their major upper airway symptoms which include sense of smell, nasal obstruction, nasal discharge, and facial pain and achieve a documented minimal clinical important difference (MCID) using a validated disease specific questionnaire. Patients should be evaluated every 6 months in the first two years of biologic initiation and every 1 year thereafter	Mean = 6.83 (Neutral), Median = 7, Mode = 7 Fleiss’ Kappa = 0.520 (Moderate agreement) Total voters = 30	Fleiss’ Kappa = 0.520 (Moderate agreement)	Revised and re-assessed in Round 3 of the Modified Delphi Process
16	Patients who are prescribed biologics to manage co-existing CRSwNP and severe asthma should achieve the patient’s and physician’s goals of treatment for both the upper and lower airway respectively. If not, clinicians should discuss with one another if another biologic or treatment strategy is needed	Mean = 6.72 (Agree), Median = 7, Mode = 7 Total voters = 30	Fleiss’ Kappa = 0.597 (Moderate agreement)	Revised and re-assessed in Round 3 of the Modified Delphi Process
17	A different biologic can be offered to a patient who fails to respond to one biologic or experiences side effects, but continues to meet inclusion criteria for another. Obtaining biologic markers such as serum IgE and eosinophils as well as FeNO (if available) may help a clinician pick the next appropriate biologic to use	Mean = 6.45 (Neutral), Median = 7, Mode = 7 Total voters = 30	Fleiss’ Kappa = 0.572 (Moderate agreement)	Revised and re-assessed in Round 3 of the Modified Delphi Process
18	CRSwNP and asthma patients who have exhausted biologic switching and not achieved adequate response in either the upper or lower airways should be first evaluated for possible revision sinus surgery. If surgery is not indicated, the patient may be started on dual biologic therapy that is best suited for the sinuses and lungs independent of each other. These decisions may be best done in multidisciplinary clinics. Dual biologic response should be evaluated at 24 weeks. If there are subjective improvements achieved in the sinuses and lungs, dual biologic therapy may be continued and re-evaluated every 6 months	Mean = 5.24 (Neutral), Median = 5, Mode = 5 Total voters = 30	Fleiss’ Kappa = 0.533 (Moderate agreement)	Revised and re-assessed in Round 3 of the Modified Delphi Process
19	The risk of side effects is low in the short-term use of biologics (12 months) in CRSwNP. However, there have been reports of adverse events with various biologics which include eosinophilic pneumonia, enthesitis/arthritis and serum sickness	Mean = 6.69 (Neutral), Median = 7, Mode = 7 Total voters = 30	Fleiss’ Kappa = 0.611 (Substantial agreement)	Revised and re-assessed in Round 3 of the Modified Delphi Process
20	Cost and access to biologics matters in the decision making of the use of biologics for CRSwNP patients with or without another Type 2 inflammatory condition. Patient preference should also be considered when considering initiation of biologics	Mean = 6.69 (Neutral), Median = 7, Mode = 7 Total voters = 30	Fleiss’ Kappa = 0.611 (Substantial agreement)	Revised and re-assessed in Round 3 of the Modified Delphi Process

**Table 5:** Round 3 of the modified Delphi process for the consensus statements for use of biologics in upper airway disease

	Statement	Descriptive Statistics	Inter-rater Reliability	Decision
1	Patients with chronic symptoms of upper airway disease which include facial pressure/pain, nasal obstruction/congestion, nasal discharge or a loss of smell should be evaluated for upper airway disease	Mean 2.94, Median 3, Mode 3 Total voters 32	Fleiss’ Kappa = 0.84 (Perfect Agreement)	Revised and included in guidelines
2	Patients with asthma and chronic symptoms of upper airway disease despite appropriate therapy should be referred for further evaluation of upper airway disease	Mean 2.69, Median 3, Mode 3 Total voters 27	Fleiss’ Kappa = 0.82 (Perfect Agreement)	Revised and included in guidelines
3	Clinician(s) evaluating for upper airway disease should evaluate the nose with nasal endoscopy or in communities where no nasal endoscopy is available, anterior rhinoscopy is acceptable when the diagnosis of nasal polyps is apparent	Mean 2.68, Median 3, Mode 3 Total voters 27	Fleiss’ Kappa = 0.81 (Pefect Agreement)	Revised and included in guidelines
4	If nasal endoscopy is unremarkable or unavailable, a CT scan could be ordered to rule out sinus disease without polyps	Mean 2.77, Median 3, Mode 3 Total voters 22	Fleiss’ Kappa = 0.78 (Substantial Agreement)	Revised and included in guidelines
5	All endotypes of CRSwNP confirmed by endoscopy or anterior rhinoscopy are considered eligible for a trial of biologic therapy	Mean 2.44, Median 3, Mode 3 Total voters 25	Fleiss’ Kappa = 0.71 (Substantial Agreement)	Revised and included in guidelines
6	A Nasal Polyp Score (NPS) of 5 is required to be considered eligible to biologic therapy	Mean 1.30, Median 1, Mode 1 Total voters 23	Fleiss’ Kappa = 0.79 (Substantial Agreement)	Removed
7	CT reports indicating polyps are not sufficient to make the diagnosis of CRSwNP and starting on biologics	Mean 2.76, Median 3, Mode 3 Total voters 25	Fleiss’ Kappa = 0.83 (Perfect Agreement)	Revised and included in guidelines
8	Biologics should be principally considered for those who have undergone adequate sinus surgery within the past 5 years and are refractory to oral and nasal steroids	Mean 2.52, Median 3, Mode 3 Total voters 25	Fleiss’ Kappa = 0.69 (Substantial Agreement)	Revised and included in guidelines
9	Patients unfit for surgery who have failed medical therapy may also be considered candidates for biologic therapy based on shared patient decision making	Mean 2.83, Median 3, Mode 3 Total voters 24	Fleiss’ Kappa = > 0.84 (Perfect Agreement)	Revised and included in guidelines
10	The adequacy of previous surgery matters in determining if subsequent surgical management is required versus initiation of biologic therapy. This should be evaluated with a CT scan and endoscopy to determine if each of the diseased sinus cavities can receive appropriate topical drug delivery	Mean 2.90, Median 3, Mode 3 Total voters 21	Fleiss’ Kappa = 0.91 (Perfect Agreement)	Revised and included in guidelines
11	Patients with CRSwNP do not need co-existing Type 2 inflammatory condition such as asthma to be considered for biologic therapy	Mean 2.90, Median 3, Mode 3 Total voters 21	Fleiss’ Kappa = 0.91 (Perfect Agreement)	Revised and included in guidelines
12	For most patients, CRSwNP symptoms need to be severe based on the clinician’s choice of a validated patient reported outcome measure (PROM) for chronic sinus disease to warrant the use of biologics	No vote required	N/A	Included in guidelines
13	There are a subgroup of patients that may score lower than severe disease on a patient-reported outcome measure (PROM) due to acclimatization to their symptoms (i.e. allergic fungal rhinosinusitis and chronic prednisone users) and these cases should be considered for biologics based on shared decision making	Mean 2.84, Median 3, Mode 3 Total voters 31	Fleiss’ Kappa = 0.86 (Perfect Agreement)	Revised and included in guidelines
14	All CRSwNP patients with lower respiratory symptoms who have not previously been evaluated for asthma should be evaluated for possible asthma and referred to a clinician who can provide a systematic evaluation	No vote required	N/A	Included in guidelines
15	In a patient with CRSwNP qualifying for biologic therapy and severe asthma, a consultation with a specialist who can manage asthma is recommended before choosing the most appropriate biologic	No vote required	N/A	Included in guidelines
16	There is insufficient evidence to make a recommendation for providing biologics to patients with CRSsNP	No vote required	N/A	Included in guidelines
17	Patients with asthma or any other type 2 conditions in the setting of CRSsNP can be considered for biologics use outside of clinical research trials, for those conditions other than CRSsNP, if they meet eligibility criteria for biologic therapy for another type 2 condition based on their respective Canadian guidelines	Mean 1.67, Median 1, Mode 1 Total voters 22	Fleiss’ Kappa = 0.73 (Substantial Agreement)	Removed
18	Biologics should not be provided to those with recurrent acute bacterial sinusitis without CRSwNP	No vote required	N/A	Removed
19	Patients with refractory CRSwNP after surgery should be counselled regarding their options which include revision sinus surgery or biologics. Referral to a sub-specialist that can counsel and/or perform extended surgical procedures should be sought	Mean 2.90, Median 3, Mode 3 Total voters 31	Fleiss’ Kappa = 0.91 (Perfect Agreement)	Revised and included in guidelines
20	Where possible, patients with Aspirin Exacerbated Respiratory Disease (AERD) should be preferentially managed by a multidisciplinary team	Mean 2.66, Median 3, Mode 3 Total voters 22	Fleiss’ Kappa = > 0.60 (Substantial Agreement)	Revised and included in guidelines
21	Patients with CRSwNP who must wait longer than 6 months for undergoing primary sinus surgery should be allowed to initiate biologic therapy as a bridge to surgical management	Mean 1.23, Median 1, Mode 1 Total voters 24	Fleiss’ Kappa = 0.74 (Substantial Agreement)	Removed
22	If a patient achieves desired symptom control on biologics prior to surgery, a patient may choose not to do surgery and continue with biologics	Mean 1.23, Median 1, Mode 1 Total voters 24	Fleiss’ Kappa = 0.74 (Substantial Agreement)	Removed
23	Biologics can be uniquely considered for hyposmic patients where their sense of smell function is required for safety reasons or for their job if they have a history CRSwNP treated with surgery with adequate control of their disease and no evidence of polyps on endoscopy. Objective testing must be performed (UPSIT $\le 33$ or Sniffin’ Sticks Test $\le$ 30)	No vote required	N/A	Removed
24	At the time of writing, there are no biological markers required to start CRSwNP patients on biologics nor any markers to indicate best biologic to use	Mean 2.68, Median 2, Mode 2 Total voters 20	Fleiss’ Kappa = 0.76 (Substantial Agreement)	Revised and included in guidelines
25	Nasal response to biologics should be assessed by 16 weeks after initiating therapy	Mean 3, Median 3, Mode 3 Total voters 21	Fleiss’ Kappa = 1.0 (Perfect Agreement)	Revised and included in guidelines
26	Patients should experience an improvement and achieve a documented minimal clinical important difference (MCID) using a validated disease specific questionnaire or the biologic should be re-evaluated	Mean 2.58, Median 3, Mode 3 Total voters 19	Fleiss’ Kappa = 0.69 (Substantial Agreement)	Revised and included in guidelines
27	Patients should be evaluated every 6 months in the first two years of biologic initiation and every 1 year thereafter	Mean 2.79, Median 3, Mode 3 Total voters 19	Fleiss’ Kappa = 0.79 (Substantial Agreement)	Revised and included in guidelines
28	When treating co-existing CRSwNP and asthma, an attempt should be made to obtain optimal results with a single biologic in both diseases	Mean 3, Median 3, Mode 3 Total voters 18	Fleiss’ Kappa = 1.0 (Perfect Agreement)	Revised and included in guidelines
29	Pre-biologic criteria may be used to qualify a patient for a second or subsequent biologic therapies in case of sub-optimal response to the first biologic	Mean 3, Median 3, Mode 3 Total voters 18	Fleiss’ Kappa = 1.0 (Perfect Agreement)	Revised and included in guidelines
30	In case of failure to respond to biologic therapy in the case of nasal polyps, obtaining biologic markers may help a clinician pick the next appropriate biologic to use	Mean 1.42, Median 1, Mode 1 Total voters 12	Fleiss’ Kappa = 0.63 (Substantial Agreement)	Removed
31	CRSwNP who have exhausted biologics and not achieved simultaneous adequate response in both the upper and lower airways could be evaluated for possible revision sinus surgery	Mean 2.54, Median 3, Mode 3 Total voters 13	Fleiss’ Kappa = 0.71 (Substantial Agreement)	Revised and included in guidelines
32	CRSwNP and asthma patients who have exhausted biologic switching and not achieved simultaneous adequate response in both the upper and lower airways and in which surgery is not indicated may be started on dual biologic therapy that is best suited for the sinuses and lungs independent of each other. These decisions may be best done in multidisciplinary clinics (MDC) or if MDC not available, in consult with other specialists taking care of this patient	Mean 1.29, Median 1, Mode 1 Total voters 14	Fleiss’ Kappa = 0.76 (Substantial Agreement)	Removed
33	The risk of side effects is low in the short-term use of biologics in CRSwNP	Mean 2.29, Median 3, Mode 3 Total voters 14	Fleiss’ Kappa = 0.66 (Substantial Agreement)	Revised and included in guidelines
34	Cost and access to biologics should be considered in the decision making of the use of biologics for CRSwNP patients with or without another Type 2 inflammatory condition	Mean 2.53, Median 3, Mode 3 Total voters 15	Fleiss’ Kappa = 0.71 (Substantial Agreement)	Revised and included in guidelines
35	Patient preference should also be considered when considering initiation of biologics	Mean 1.14, Median 1, Mode 1 Total voters 14	Fleiss’ Kappa = 0.93 (Perfect Agreement)	Removed

## Abbreviations

CRS: Chronic rhinosinusitis
CRSwNP: Chronic rhinosinusitis with nasal polyposis
ESS: Endoscopic Sinus Surgery
IL: Interleukin
Ig: Immunoglobulin
CRSsNP: Chronic rhinosinusitis without nasal polyposis
SNOT-22: Sino-Nasal Outcome Test-22
CSS: Chronic sinusitis survey
RSDI: Rhinosinusitis disability index
VAS: Visual analogue scale
CAD: Canadian
NPIF: Nasal peak inspiratory flow
Serum ECP: Serum eosinophil cationic protein
IL-5Rα: Interleukin-5 receptor α
MPO: Myeloperoxidase
MCID: Minimal Clinically Important Difference
SF-36: 36-Item Short Form Survey
UPSIT: The University of Pennsylvania Smell Identification Test
RSOM-31: 31-Item Rhinosinusitis Outcome Measure
AQLQ: Asthma Quality of Life Questionnaire
FEV1: Forced Expiratory Volume
PEF: Peak Expiratory Flow
EQ-5D: Generic health-related quality of life questionnaire
ACQ-6: 6-Question Asthma Control Questionnaire
ACQ-5: 5-Question Asthma Control Questionnaire
LMK: Lund-Mackay Score
zLMK: Zinreich-modified Lund–Mackay
TSLP: Thymic stromal lymphopoietin
OCS: Oral corticosteroid
